# Novel ultrasonographic Halo Score for giant cell arteritis: assessment of diagnostic accuracy and association with ocular ischaemia

**DOI:** 10.1136/annrheumdis-2019-216343

**Published:** 2020-01-03

**Authors:** Kornelis S M van der Geest, Frances Borg, Abdul Kayani, Davy Paap, Prisca Gondo, Wolfgang Schmidt, Raashid Ahmed Luqmani, Bhaskar Dasgupta

**Affiliations:** 1 Rheumatology and Clinical Immunology, University Medical Center Groningen, University of Groningen, Groningen, The Netherlands; 2 Rheumatology, Southend University Hospital NHS Foundation Trust, Westcliff-on-Sea, Essex, UK; 3 Rehabilitation Medicine, University Medical Center Groningen, University of Groningen, Groningen, The Netherlands; 4 Medical Centre for Rheumatology Berlin-Buch, Immanuel-Krankenhaus GmbH, Berlin, Germany; 5 Rheumatology, University of Oxford Nuffield Department of Orthopaedics Rheumatology and Musculoskeletal Sciences, Oxford, Oxfordshire, UK

**Keywords:** giant cell arteritis, ultrasonography, systemic vasculitis

## Abstract

**Objectives:**

Ultrasound of temporal and axillary arteries may reveal vessel wall inflammation in patients with giant cell arteritis (GCA). We developed a ultrasound scoring system to quantify the extent of vascular inflammation and investigated its diagnostic accuracy and association with clinical factors in GCA.

**Methods:**

This is a prospective study including 89 patients suspected of having GCA, of whom 58 had a confirmed clinical diagnosis of GCA after 6 months follow-up. All patients underwent bilateral ultrasound examination of the three temporal artery (TA) segments and axillary arteries, prior to TA biopsy. The extent of vascular inflammation was quantified by (1) counting the number of TA segments and axillary arteries with a halo and (2) calculating a composite Halo Score that also incorporated the thickness of each halo.

**Results:**

Halo counts and Halo Scores showed moderate diagnostic accuracy for a clinical diagnosis of GCA. They correlated positively with systemic inflammation. When compared with the halo count, the Halo Score correlated better with C-reactive protein (CRP) levels and allowed to firmly establish the diagnosis of GCA in more patients. Higher halo counts and Halo Scores were associated with a higher risk of ocular ischaemia. They allowed to identify subgroups of patients with low risk (≤5%) and high risk of ocular ischaemia (>30%).

**Conclusions:**

Ultrasound halo scoring allows to quantify the extent of vascular inflammation in GCA. Extensive vascular inflammation on ultrasound may provide strong diagnostic confirmation and associates with ocular ischaemia in GCA.

**Video abstract:**

**Key messages**

**What is already known about this subject?**

- Vascular ultrasound is the recommended first-line investigation in the diagnostic work-up of giant cell arteritis (GCA).

**What does this study add?**

- This prospective study shows that the extent of vascular inflammation on ultrasound, that is, a halo count ≥2 or Halo Score ≥3, identifies GCA patients at high risk for ocular ischaemia (>30%). Patients below these cut-off points have a low risk for ischaemic vision loss (≤5%). Furthermore, halo counts and Halo Scores associate with systemic inflammation.
- The presence of a high Halo Score ≥10 may help to firmly diagnose GCA in a substantial portion of patients, that is, with high specificity (>95%) and a high positive likelihood ratio (>5.0).

**How might this impact on clinical practice or future developments?**

- Quantifying the extent of vascular inflammation by ultrasound has diagnostic value and may help to discriminate between patients with a high or low risk for ocular ischaemia.
- The halo count and Halo Score await further validation and could potentially be interesting outcome parameters in translational and therapeutic studies.

## Introduction

Giant cell arteritis (GCA) is an autoimmune disease characterised by inflammation of large-sized and medium-sized arteries. Ocular ischaemia is a feared complication of GCA.^1^ Laboratory testing often reveals systemic inflammation, that is, high C-reactive protein (CRP) levels, anaemia and thrombocytosis.^2^

EULAR recommendations identify temporal and axillary artery ultrasound as the first-line investigation in patients suspected of having GCA.^3^ A halo is the main ultrasound finding suggestive of GCA.^4 5^ A halo is a homogeneous, hypoechoic wall thickening of the artery, reflecting inflammation-induced oedema of the arterial wall.^6^ Ultrasound has a 77% sensitivity and 96% specificity for GCA.^7^

Little is known about the relationship between the extent of vascular inflammation on ultrasound and disease severity in GCA. Aschwanden *et al* evaluated 11 vascular regions for the presence of a halo and showed that involvement of large systemic arteries is associated with more weight loss.^8^ Schmidt *et al* linked axillary artery involvement to a low risk of eye complications.^9^ The risk of eye complications was not related to the number of temporal artery (TA) segments with a halo in the latter study. In neither of these studies was halo thickness incorporated into the analysis of disease extent.

In the current study, we evaluated whether the extent of vascular inflammation on ultrasound could be linked to disease severity in GCA. To enumerate disease extent, we first calculated the number of TA segments and axillary arteries with a halo sign. Furthermore, we developed a novel Halo Score that encompassed both the number of halos and the maximum halo thickness in each vascular region. We investigated the diagnostic accuracy of halo counts and Halo Scores, and their relationship with disease severity as indicated by ocular ischaemia and systemic inflammation.

## Methods

### Patients

Consecutive patients suspected of having GCA (n=104) were prospectively recruited at the Rheumatology Department of Southend University Hospital between June 2010 and December 2013 as part of the TABUL study.^10^ Patients underwent arterial ultrasound followed by a TA biopsy (TAB). Ultrasound and TAB were performed within 7 days after initiation of high-dose glucocorticoids. Patients were re-assessed after 6 months. The reference standard for GCA was the final clinical diagnosis after 6 months (online supplementary methods).

10.1136/annrheumdis-2019-216343.supp1 Supplementary data

### Ultrasound

Ultrasound scans were performed by a single, experienced ultrasonographer (BD) with an Esaote MyLab70 or MyLabTwice. A linear probe (LA435) with a grey-scale frequency of 18 MHz and colour Doppler frequency of 9 MHz was used. The focus was positioned at 5 mm below the skin for the TA. The pulse repetition frequency was 2–3 kHz. The colour box was set at an angle of at least 60°. The gain setting was adjusted to just fill the lumen. Patients were lying in a (semi-)recumbent position during the examination. The common superficial TA, its parietal and frontal branches, as well as the axillary arteries were fully and bilaterally examined in the long and short planes. In each vascular territory, the thickness of the largest halo was measured with one decimal place at the point of maximum thickness in the longitudinal plane. The ultrasonographer was not blinded to the clinical data of the patient. An ultrasound expert panel evaluated all scans and reports to monitor the scan quality and the adequacy of the reported findings. A halo sign was morphologically defined as an ultrasound finding of a dark hypoechoic area around the vessel lumen. A composite Halo Score was developed based on percentiles of halo thickness in patients with GCA.

### Statistics

Information regarding statistics is provided in the figure legends and online supplementary methods. P values <0.05 were considered statistically significant. Data were analysed with IBM SPSS Statistics V.25, StatsDirect V.3.1.22 and Graphpad Prism V.5.

## Results

### Patient characteristics

Out of 104 patients with suspected GCA, 92 patients underwent both ultrasound and TAB at baseline, and 89 patients completed 6 months follow-up (table 1). A clinical diagnosis of GCA was established in 58 out of 89 patients. Diagnoses in non-GCA patients are shown in online supplementary table S1.

10.1136/annrheumdis-2019-216343.supp2 Supplementary data

**Table 1:** Patients’ characteristics

Patients’ characteristics	All patients (n=89)	Patients with GCA (n=58)	Patients without GCA (n=31)
Sex, no. of males	26 (29%)	15 (26%)	11 (36%)
Age, median (range) years	73 (44–96)	74 (50–96)	67 (44–90)
High-dose steroids started ≤7 days before baseline, no. of patients	75 (84%)	49 (85%)	26 (84%)
TAB positive according to pathologist, no. of patients	26 (29%)	26 (45%)	0 (0%)
TAB length, median (range) mm	7 (2–20)	7 (2–20)	8 (2–13)
Fulfilling 1990 ACR criteria for GCA, no of patients	72 (81%)	50 (86%)	22 (71%)
Any head pain present, no of patients	85 (96%)	55 (95%)	30 (97%)
New localised head pain, no of patients	77 (87%)	48 (83%)	29 (94%)
New generalised scalp tenderness, no of patients	52 (58%)	35 (60%)	17 (55%)
Swelling over temporal artery, no of patients	22 (25%)	14 (24%)	8 (26%)
Pain over temporal artery, no of patients	49 (55%)	29 (50%)	20 (65%)
Jaw claudication, no of patients	42 (47%)	32 (55%)	10 (32%)
Tongue claudication, no of patients	3 (3%)	2 (3%)	1 (3%)
Any visual symptoms, no of patients	47 (53%)	30 (52%)	17 (55%)
Reduced or lost vision, no of patients	38 (43%)	26 (45%)	12 (39%)
Double vision, no of patients	9 (10%)	4 (7%)	5 (16%)
Amaurosis fugax, no of patients	2 (2%)	2 (3%)	0 (0%)
Anorexia, no of patients	31 (35%)	22 (38%)	9 (29%)
Fatigue, no of patients	65 (73%)	42 (72%)	23 (74%)
Fever or night sweats, no of patients	38 (43%)	25 (43%)	13 (42%)
Polymyalgia, no of patients	16 (18%)	14 (24%)	2 (7%)
Temporal artery thickening, no of patients	28 (32%)	21 (36%)	7 (23%)
Temporal artery tenderness, no of patients	50 (56%)	29 (50%)	21 (68%)
Temporal artery abnormal pulse, no of patients	18 (20%)	16 (28%)	2 (7%)
Axillary artery tenderness, no of patients	8 (9%)	5 (9%)	3 (10%)
AION*, no of patients	15 (17%)	10 (17%)	5 (16%)
PION*, no of patients	5 (6%)	2 (3%)	3 (10%)
RAPD*, no of patients	7 (8%)	5 (9%)	2 (7%)
Ocular ischaemia (AION/PION/RAPD), no of patients	19 (21%)	12 (21%)	7 (23%)
Ocular palsy*†, no of patients	0 (0%)	0 (0%)	0 (0%)
Bruits*, no of patients	0 (0%)	0 (0%)	0 (0%)
Stroke*	2 (2%)	0 (0%)	2 (7%)
ESR, mm/hour,† median (range)	34 (3–90)	44 (3–90)	9 (3–77)
CRP, mg/L,† median (range)	46 (3–329)	54 (3–329)	13 (3–205)
Haemoglobin (g/dL), median (range)	12.8 (8.9–16.0)	12.0 (8.9–15.5)	13.5 (10.1–16.0)
Platelets, 10^9^/L, median (range)	343 (126–661)	363 (167–661)	317 (126–522)

Details of the 89 patients recruited in the TABUL study at Southend University Hospital, who underwent ultrasound, temporal artery biopsy and 6 months follow-up.

ESR was determined in n=57 patients and CRP in n=54 subjects. ESR and CRP were measured before initiation of high-dose glucocorticoid treatment. Haemoglobin levels and platelet counts were determined prior to high-dose glucocorticoid treatment or within 7 days after initiation of this treatment.

*Considered negative if not reported.

†ESR and CRP were not performed in every subject.

AION, anterior ischaemic optic neuropathy;CRP, C-reactive protein; ESR, erythrocyte sedimentation rate; GCA, giant cell arteritis; PION, posterior ischaemic optic neuropathy; RAPD, relative afferent pupillary defect; TAB, temporal artery biopsy.

### Halo thickness and construction of the Halo Score

**Figure 1:**
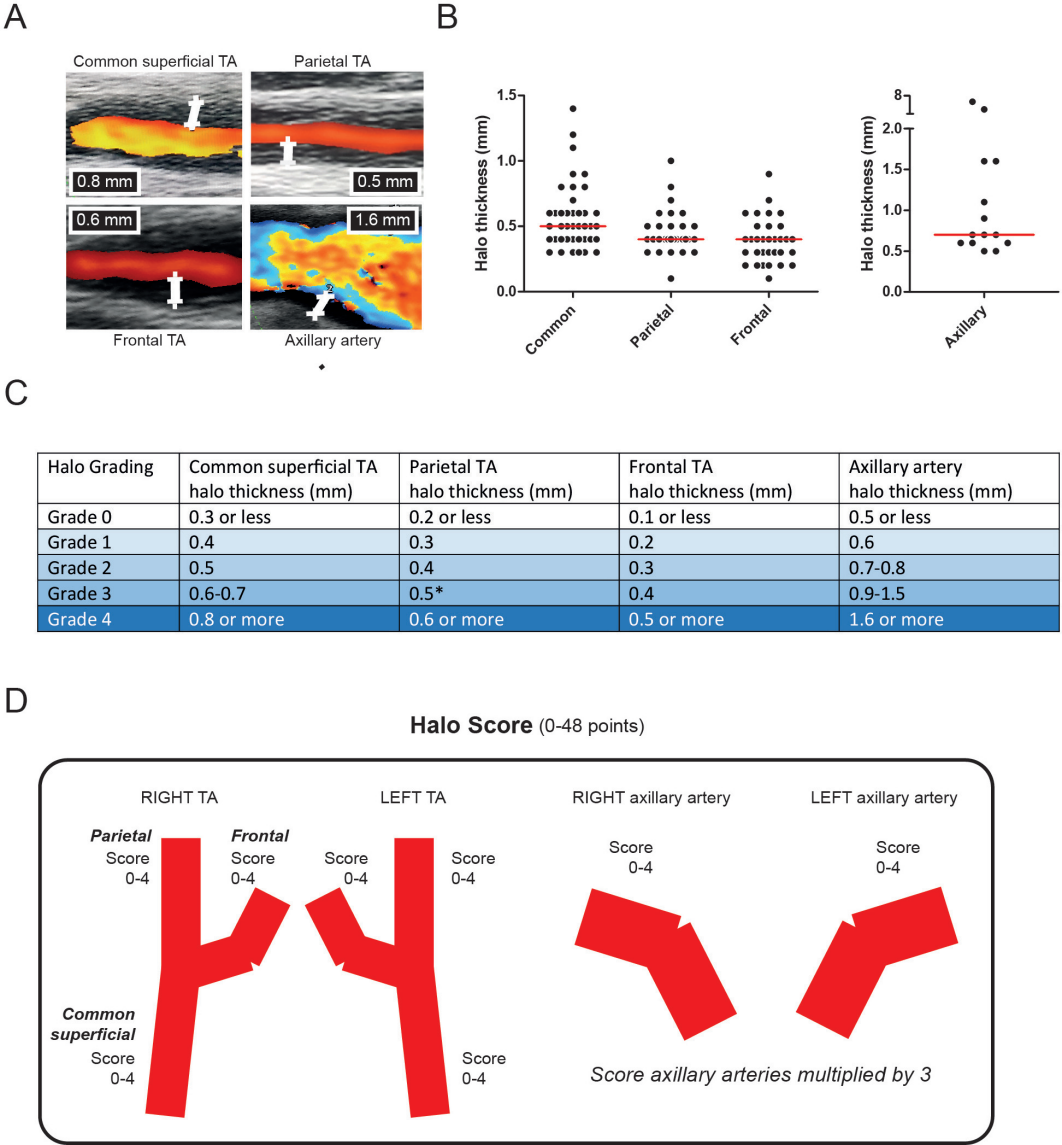
Ultrasound halo scoring. (A) Representative ultrasound images of halo signs, and measurements of halo thickness, in the common superficial TA, parietal TA, frontal TA and axillary artery. (B) Thickness of halo signs that were reported in 41 common TA segments, 29 parietal TA segments, 32 frontal TA segments and 14 axillary arteries of patients with GCA. (C) Halo grade scoring system and cut-off values. Due to similar 40% and 60% percentile boundaries in the parietal TA, a cut-off value of 0.5 mm was used for a grade 3 halo in this TA segment. (D) Construction of the Halo Score. Axillary artery scores were multiplied by 3 to give equal weight to the TA and axillary artery for the Halo Score. TA, temporal artery.

At baseline, the three TA segments and the axillary artery were examined by ultrasound on each side. In GCA patients, halos were reported in 41 common TA segments, 29 parietal TA segments, 32 frontal TA segments and 14 axillary arteries (figure 1A). If present, the maximum thickness of the halo was measured (figure 1B).

In order to develop a composite Halo Score, we identified the 20%, 40%, 60% and 80% percentiles of the maximum halo thickness in the TA segments and axillary arteries of patients with GCA (online supplementary table S2). Based on these arbitrary percentiles, we assigned halo grade scores to each TA segment and axillary artery (figure 1C). The distribution of halo grades among GCA patients with a halo is shown in online supplementary tables S3 and S4.

The sum of all halo grade scores was used to construct the Halo Score for each patient (figure 1D). To give equal weight to temporal and axillary arteries, the halo grade scores of the axillary arteries were multiplied by a factor of 3. Therefore, the Halo Score values could range from 0 to 48. For halo counts in TA segments and axillary arteries, no correction factor was used for the axillary artery. Thus, halo counts could vary from 0 to 8.

### Diagnostic accuracy for GCA

**Figure 2:**
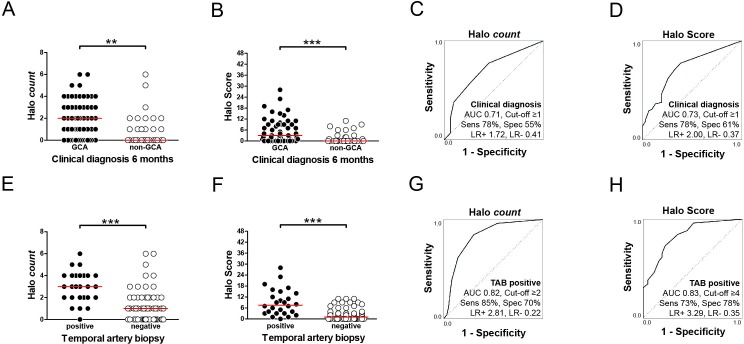
Diagnostic accuracy of halo count and Halo Score for GCA. (A) Baseline halo count in common superficial TAs, parietal TAs, frontal TAs and axillary arteries and (B) Halo Scores in patients with an eventually confirmed clinical diagnosis of GCA (n=58) versus non-GCA patients (n=31). (C) ROC curve showing the diagnostic accuracy of baseline halo counts and (D) Halo Scores for an eventual clinical diagnosis of GCA after 6 months. The optimal cut-off points were determined by Youden index. (E) Baseline halo counts and (F) Halo Scores in patients with a positive TAB (n=26) versus patients with a negative TAB (n=63). Overall, 25 TABs showed transmural inflammation and/or giant cells. One TAB considered positive for GCA showed an adventitial infiltrate, elastic lamina disruption and intimal hyperplasia without transmural inflammation/giant cells. (G) ROC curve showing the diagnostic accuracy of halo counts and (H) Halo Scores for a positive TAB. The optimal cut-off point was determined by Youden index. AUC, area under the curve; LR+, positive likelihood ratio; LR−, negative likelihood ratio; ROC, receiver operating characteristic; Sens, sensitivity; Spec, specificity; TA, temporal artery; TAB, temporal artery biopsy. Statistical significance at (A, B, E, F) was tested by Mann-Whitney U test: **p<0.01, ***p<0.001.

Baseline halo counts and Halo Scores were higher in patients with a subsequently confirmed diagnosis of GCA than patients without GCA (figure 2A, B). Two non-GCA patients showed a high halo count. These halos were small in one male patient and could be attributed to atherosclerosis in one female patient (online supplementary table S5). Halo counts and Halo Scores showed similar diagnostic accuracy for a clinical diagnosis of GCA, as indicated by an area under the curve (AUC) of >0.70 in the receiver operating characteristic (ROC) curve analysis (figure 2C, D). At the optimal cut-off point, the sensitivity/specificity and likelihood ratios were comparable for both ultrasound parameters. In a subanalysis restricted to halo counts/Halo Scores in the TA only, similar diagnostic accuracy was obtained (online supplementary table S6).

Alternative cut-off points providing a specificity of 95% for a clinical diagnosis of GCA could be obtained: a halo count of ≥6, or Halo Score of ≥10. Although a Halo Score of ≥10 was present in 12 patients (21% of all patients with GCA), only two patients (3% of all patients with GCA) showed a halo count of ≥6 (online supplementary table S7). The positive likelihood ratio of a Halo Score ≥10 was high (LR +6.41), but poor for the halo count at this cut-off point (LR +1.07). Thus, the Halo Score could be used more effectively than the halo count to establish a diagnosis of GCA in more patients.

### Diagnostic accuracy for positive TAB

The frequency and thickness of halos was higher in GCA patients with a positive TAB than patients with a negative TAB (online supplementary table S8). Consequently, halo counts and Halo Scores were higher in patients with a positive TAB than those with a negative biopsy (figure 2E, F). Both ultrasound parameters showed a good ability to predict a positive TABwith an AUC >0.80 in the ROC analysis (figure 2G, H). The sensitivity and specificity, positive likelihood ratios>2 and negative likelihood ratios<0.5 indicated that halo counts and Halo Score may help to predict the TAB result. Comparable diagnostic accuracy was obtained, if only TAs halo counts were taken into account (online supplementary table S6).

### Effect of glucocorticoid treatment

Halo signs may disappear within days to weeks following initiation of glucocorticoid treatment.^5 11^ When comparing patients with GCA receiving glucocorticoids for 0–1 days, 2–3 days and 4–7 days prior to ultrasound, we did not observe any differences in halo counts or Halo Scores (online supplementary figure S1). Patients using glucocorticoids for 4–7 days prior to ultrasound tended to have a higher prevalence of ocular ischaemia and polymyalgic symptoms when compared with other patients, although not statistically significant (online supplementary table S9).

### Vascular and systemic inflammation

**Figure 3:**
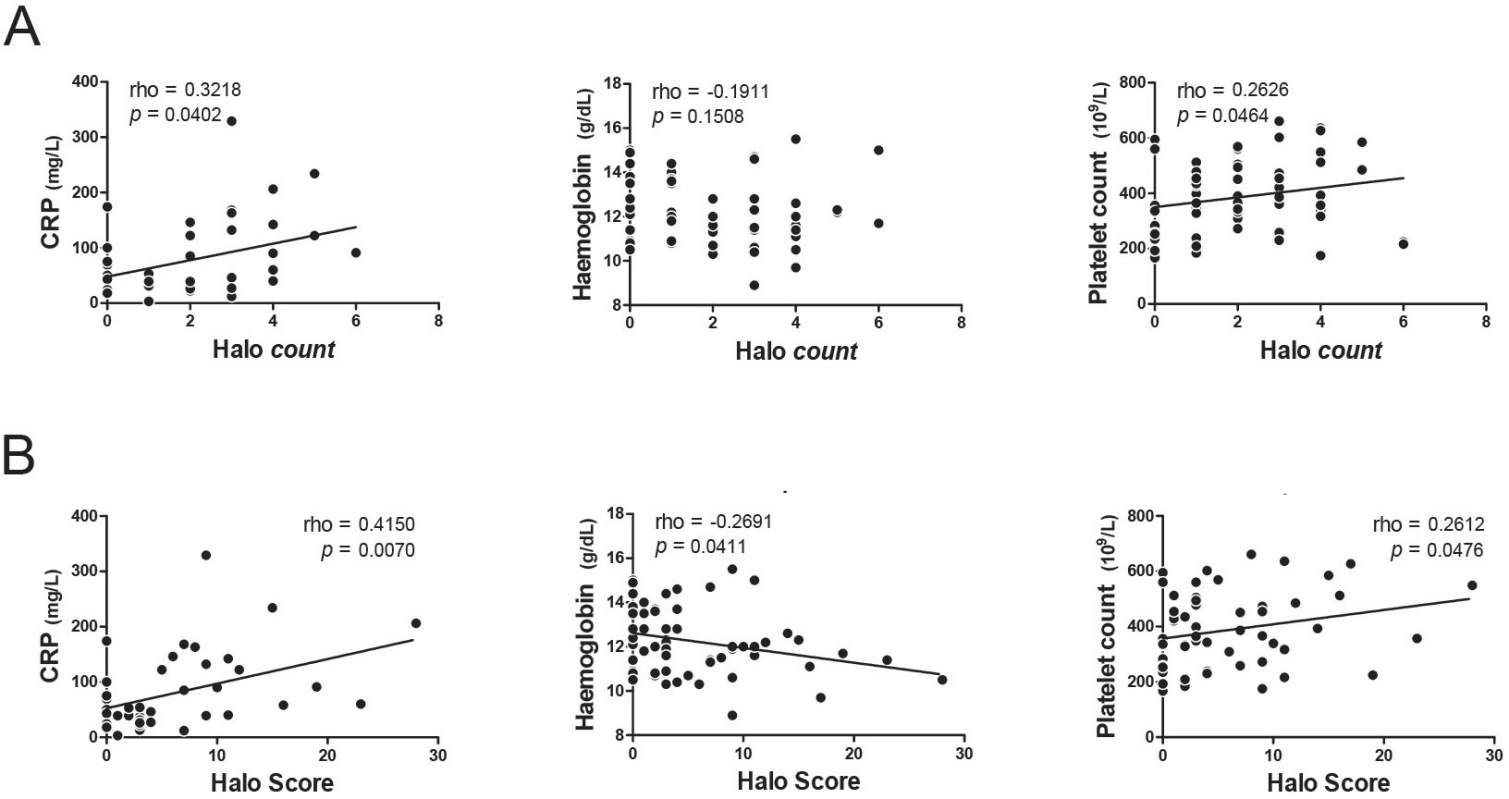
Relationship of halo count and Halo Score with systemic inflammation. (A) Correlation of halo counts and (B) Halo Scores with CRP, haemoglobin and platelets in patients with a clinical diagnosis of GCA. CRP levels were determined prior to initiation of treatment in 41 GCA patients. Haemoglobin levels and platelet counts were measured prior to treatment or within 7 days after initiation of high-dose glucocorticoids in 58 GCA patients. Correlations were determined by Spearman’s rank correlation coefficient.

We questioned if ultrasound findings could be linked to systemic inflammation in patients with GCA. Halo counts showed no correlation with haemoglobin levels but correlated positively with CRP levels and platelet counts (figure 3A). The Halo Score correlated even better with CRP levels, showed a positive correlation with platelets counts, and correlated negatively with haemoglobin levels (figure 3B). In a subanalysis of halo counts/Halo Scores restricted to the TA only, these correlations became less clear (online supplementary table S10). The presence of axillary artery involvement tended to be associated with more systemic inflammation (online supplementary table S11). Taken together, Halo Scores associated stronger with systemic inflammation than halo counts.

The erythrocyte sedimentation rate (ESR), as measured by a capillary photometric-kinetic technique (Alifax),^12^ was remarkably low in patients with GCA (online supplementary table S12). Pretreatment ESR was <30 mm/hour in 31% of GCA patients, while CRP was <10 mg/L in 2% of patients. Only 46% of GCA patients showed an ESR >50 mm/hour. Thus, the ESR showed no correlation with CRP levels, halo counts or Halo Scores (online supplementary figure S2).

### Extent of vascular inflammation and patients’ characteristics

We performed multiple linear regression analysis to investigate if ocular ischaemia, or perhaps other clinical characteristics, were associated with higher halo counts or Halo Scores. Ocular ischaemia was defined by the presence of anterior ischaemic optic neuropathy (AION), posterior ischaemic optic neuropathy (PION) and/or a relative afferent pupillary defect (RAPD). Both ocular ischaemia and male sex were independently associated with higher halo counts and Halo Scores in patients with GCA (table 2). No further relationships were observed between clinical features and ultrasound parameters.

**Table 2:** Variables predicting the extent of vascular inflammation on ultrasound

Dependent variable	Predicting variable	Final model of multiple linear regression B (95% CI)	P value
Halo count	Age	–	
	Sex	1.109 (0.172 to 2.047)*	0.021
	Ocular ischaemia	1.103 (0.089 to 2.116)*	0.034
	Polymyalgia	–	
	Two or more systemic symptoms	–	
	Temporal artery palpable changes	–	
Halo Score	Age	–	
	Sex	2.902 (0.100 to 6.984)†	0.041
	Ocular ischaemia	3.488 (0.305 to 8.143)†	0.028
	Polymyalgia	2.813 (−0.053 to 7.080)†	0.056
	Two or more systemic symptoms	–	
	Temporal artery palpable changes	–	

Data are shown for baseline halo count and Halo Scores in patients with GCA (n=58). Multiple linear regression analysis was performed with backward exclusion of predicting variables. Since the Halo Score was not normally distributed, the Halo Score was transformed by square root. The probability of F for removal was 0.10. Results of the final model are shown. Age in years. Sex: 0=female, 1=male. Ocular ischaemia (ie, anterior ischaemic optic neuropathy, posterior ischaemic optic neuropathy and/or relative afferent pupillary defect), polymyalgia, two or more systemic symptoms (ie, anorexia, fever/night sweats, fatigue), temporal artery palpable changes (ie, thickening and/or loss of pulse): 0=absent, 1=present. (−) Variable removed due to backward exclusion.

*R^2^=0.157, F(2,55) = 5.138, p=0.009.

†R^2^=0.207, F(3,54) = 4.688, p=0.006.

GCA, giant cell arteritis.

### Diagnostic accuracy for ocular ischaemia

**Figure 4:**
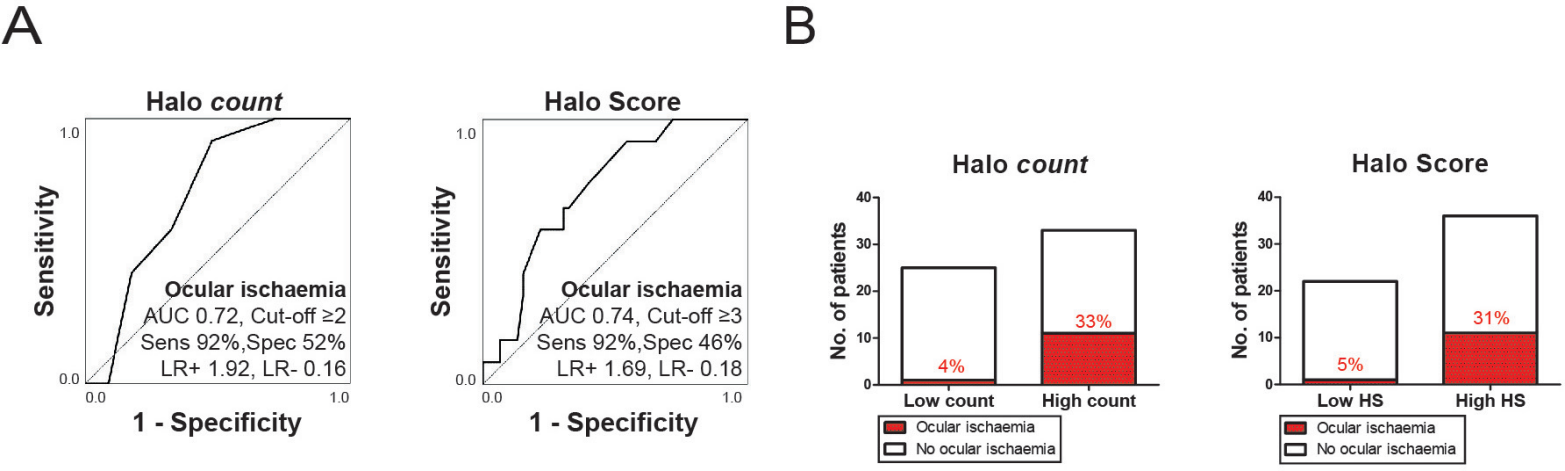
Halo count and Halo Score associated with ocular ischaemia. (A) Receiver operating characteristic curve showing diagnostic accuracy of baseline halo count (left panel) and Halo Score (right panel) for concomitant presence of ocular ischaemic symptoms. Ocular ischaemia was defined as the presence of anterior ischaemic optic neuropathy, posterior ischaemic optic neuropathy and/or a relative afferent pupillary defect. The optimal cut-off point was determined by Youden index. (B) Presence of ocular ischaemia (percentages are shown) among patients with low versus high halo count (left panel), or low versus high Halo Score (right panel) as determined by the optimal cut-off points mentioned at (A). AUC, area under the curve; HS, Halo Score; LR+, positive likelihood ratio; LR−, negative likelihood ratio; Sens, sensitivity; Spec, specificity.

Halo counts and Halo Scores showed fair ability to discriminate between GCA patients with and without ocular ischaemia, as indicated by an AUC >0.70 in the ROC analysis (figure 4A). At the optimal cut-off point for ocular ischaemia, that is, halo count ≥2 or Halo Score ≥3, an excellent sensitivity and poor specificity were obtained. At the optimal cut-off points, positive likelihood ratios <2 indicated that halo counts and Halo Scores were not helpful in predicting the presence of ocular ischaemia. However, negative likelihood ratios were <0.2. Thus, low halo counts and Halo Scores helped to identify a substantial group of patients with a low risk of ocular ischaemia (figure 4B). In a subanalysis of halo counts and Halo Scores restricted to the TA only, comparable diagnostic accuracy for ocular ischaemia was obtained (online supplementary figure S3). The presence of axillary involvement per se showed no effect on the risk of ocular ischaemia (online supplementary table S13).

Next, we evaluated if halo counts and Halo Scores were independent predictors for ocular ischaemia in a logistic regression analysis (online supplementary table S14). A halo count ≥2 provided an OR for ocular ischaemia of 12.000 (95% CI=1.430 to 100.705; p=0.022), whereas a Halo Score ≥3 showed an OR of 9.880 (95% CI=1.137 to 85.887; p=0.038). Other clinical characteristics were not predictive of ocular ischaemia. Thus, halo counts and Halo Scores were independent predictors of ocular ischaemia.

## Discussion

We show that the extent of vascular inflammation on ultrasound, as quantified by the halo count and novel Halo Score, can be linked to ocular ischaemia and systemic inflammation in GCA. The Halo Score allowed to firmly establish a diagnosis of GCA in more patients than the halo count.

The extent of inflammation was measured in the three TA segments and axillary arteries. Subclavian and facial arteries were not evaluated. However, axillary artery involvement identifies the vast majority of patients with inflammation of large systemic arteries,^13^ whereas TA involvement identifies nearly all patients with cranial artery involvement.^14^ EULAR recommendations recognise temporal and axillary artery ultrasound as the first-line investigation in GCA.^3^ Examination of temporal and axillary arteries might therefore provide a reasonable estimation of disease extent in GCA.

Extensive vascular inflammation identified GCA patients at high (>30%) risk of ocular ischaemia. However, half of patients showed low halo counts/Halo Scores and a ≤5% risk of GCA-related vision loss. As visual symptoms described by patients are not always related to GCA, we strictly defined ocular ischaemia by the presence of AION, PION and/or RAPD. Patients with suggestive eye symptoms were referred to the ophthalmologist in this single hospital study. Nevertheless, it might be a bias that not every patient underwent ophthalmological examination. Previously, no relationship was noted between the number of TA segments with a halo and ocular complications.^9^ However, the definition of ocular complications in the latter study was broader than in the current study. Wall thickening of arteries supplying the retina is thought to cause ocular ischaemia in GCA.^1 15^ Our findings indicate that wall thickening in the latter arteries likely parallels that in other arteries in GCA.

The extent of vascular inflammation correlated well with systemic inflammation in patients with GCA. Halo counts correlated positively with CRP levels and platelets counts. Halo Scores correlated even better with CRP levels than halo counts and also showed an inverse correlation with haemoglobin levels. No association was found with the ESR, which was measured by a capillary photometric–kinetic technique. This method provides an indirect estimation of the ESR^12^ and might be less accurate than the traditional Westergren in the context of rheumatic inflammatory diseases.^16 17^ Overall, our findings suggest a link between arterial and systemic inflammation in GCA.

Halo counts and Halo Scores showed comparable diagnostic accuracy for a clinical diagnosis of GCA. At the optimal cut-off points, both ultrasound parameters provided fair sensitivity (78%) but moderate specificity (55%–61%) for a diagnosis of GCA. Even better diagnostic accuracy was obtained for a positive TAB, which supports the idea that ultrasound might replace a TAB under certain conditions.^3^ Alternative cut-off points providing 95% specificity for a clinical diagnosis of GCA could also be obtained. Few patients with GCA fulfilled this cut-off point for the halo count. In contrast,>20% of patients with GCA showed Halo Scores above the 95% specificity cut-off point, that is, a score ≥10. At this cut-off point, a high positive likelihood ratio could be obtained for the Halo Score, which allowed us to make a confident diagnosis of GCA in a substantial portion of patients.

Male sex was associated with higher halo counts and Halo Scores in patients with GCA. Recently, male sex predicted the presence of a halo sign on ultrasound in patients with GCA.^18^ It might be possible that GCA is associated with more arterial thickening in men than women. However, it is also conceivable that the arterial calibre and arterial wall thickness are in general higher in men than women.^5^ It would be interesting to collect sex-specific, normative data on arterial wall thickness.

A halo was morphologically defined as a dark hypoechoic area around the vessel lumen. As the halo compression sign was reported at the end of our study,^19^ this sign was not tested. Recently, diagnostic cut-off values have been described for the intima–media thickness in TAs, as measured by a 22 MHz transducer.^20^ Although still considered state of the art, our 18 MHz transducer frequently does not allow us to visualise the intima–media complex of the TAs. Halo thickness and intima–media thickness are therefore not fully interchangeable parameters. Halo counts and thickness might have been higher if measured with higher frequency transducers. In accordance with Monti *et al*,^21^ we observed relatively similar halo thickness among the three TA segments. Halo counts in the latter study were comparable with those in the current study.

The same morphological halo definition was applied to the axillary arteries. The dark hypoechoic halo pattern differs from the normal intima–media complex, which can be readily identified as a double line in the axillary artery.^6^ Part of halos reported in the axillary artery were smaller than a recently proposed diagnostic cut-off value, that is, 1.0 mm.^20^ However, two provisional reports have suggested that axillary arteries may be inflamed despite a halo thickness <1.0 mm on ultrasound.^22 23^ Since halos <1.0 mm might still relate to disease activity, these halos were included in the halo count/Halo Score. Overall, our main study findings were not compromised by axillary artery involvement, as indicated by our subanalyses restricted to the TA only.

We observed no clear association between short-term glucocorticoid treatment and extent of vascular inflammation on ultrasound. Serial ultrasound examinations before and after initiation of glucocorticoid treatment have shown that it takes weeks to months before the majority of TA halos disappear, while only few axillary artery halos disappear.^5 11 24^ In our study, lack of treatment effect may have two explanations. First, treatment duration might have been too short. Second, patients taking glucocorticoids for 4–7 days showed a slightly higher prevalence of symptoms associated with higher halo counts/Halo Score than patients with shorter treatment duration.

A strong point of our study is its prospective design with patients undergoing ultrasound and TAB according to a fixed protocol. The clinical diagnosis was rigorously established after 6 months follow-up. Ultrasound was performed by an experienced ultrasonographer. Our study has also potential limitations. There was a bias towards cranial GCA. The ultrasonographer was not blinded to the clinical data. However, a symptom likely to bias the ultrasonographer, that is, an abnormal TA on palpation, showed no effect on halo counts or Halo Scores. The intra-rater and inter-rater reliabilities were not tested, and should be evaluated in future studies. Nevertheless, the quality of the ultrasound scans and reports was monitored by an expert panel. Our findings were derived from a post-hoc analysis of a diagnostic trial and obtained from a single centre.^10^ The Halo Score should be further validated by currently ongoing, prospective, multicentre studies (ClinicalTrials.gov NCT03765788; and NIHR Portfolio study #264294), prior to application in the clinic.

In conclusion, the extent of arterial inflammation in GCA can be quantified by ultrasound halo scoring. A high volume of vascular inflammation on ultrasound might strongly support the diagnosis of GCA and identifies patients at risk for ocular ischaemia. The clinical application of halo counts and Halo Scores warrants further validation in other studies.
