# Supplementary material for: Novel ultrasonographic Halo Score for giant cell arteritis: assessment of diagnostic accuracy and association with ocular ischaemia
Source: Ann Rheum Dis. 2020 Jan 3;79(3):393–9. doi: 10.1136/annrheumdis-2019-216343 (PMC7034352; doi:10.1136/annrheumdis-2019-216343)
Supplement: Supplementary data [file annrheumdis-2019-216343supp001.pdf]

Supplementary Methods

Reference standard for GCA

The final clinical diagnosis was made by the treating physician after 6 months follow-up. The clinical diagnosis was guided by the ACR 1990 classification criteria for GCA (including the temporal artery biopsy result)[1], the development of complications consistent with GCA during follow-up (e.g. jaw claudication, tongue claudication, ocular ischaemia), and the emergence of another disease explaining the symptoms.

The ultrasound scan was performed by an investigator (BD) other than the treating physician (FB). The ultrasonographer was neither involved in the management of the patients, nor in establishing the final clinical diagnosis. In principle, the ultrasound result was not communicated to the treating physician. However, if the treating physician planned to quickly withdraw or stop treatment within two weeks after baseline, the treating physician was made aware of the ultrasound results. In this case, it was considered unethical to withhold this information from the treating physician. The clinical judgement of the treating physician eventually determined whether knowledge of the ultrasound findings altered the diagnosis and management of the patient. More detailed information regarding the TABUL study protocol has been reported previously [2].

Statistical analysis

The Mann-Whitney U test was used for comparison of continuous variables in two independent groups. If more groups were compared, the Mann-Whitney U test was preceded by the Kruskal-Wallis test. Fisher's exact test or Chi-squared test were used for comparison of categorical variables. Correlations were determined by Spearman's rank correlation coefficient.

Receiver operating characteristic (ROC) analysis with area under the curve (AUC) was performed. Optimal cut-off points were determined according to the Youden Index. The sensitivity, specificity, positive likelihood ratio and negative likelihood ratio at the optimal cut-off points were determined.

Multiple linear regression was performed with backward exclusion of predicting variables. The probability of F for removal was 0.10. Either halo counts or Halo Scores were used as the dependent variable. Predicting variables were: age in years; sex: 0 = female, 1= male; ocular ischaemia, polymyalgia, two or more systemic symptoms, temporal artery palpable changes: 0 = absent, 1 = present. R squared (R^2) was reported. Due to non-normal distribution, Halo Scores were transformed by square root. Normality of residuals was tested by histograms and P-P plots. Linearity and homoscedasticity was tested by scatter plots and P-P plots. Multicollinearity was excluded by Pearson correlation coefficient <0.7, variance inflation factor (VIF) <10, tolerance statistics >0.2 and Collinearity Diagnostics.

Binary logistic regression was performed with backward exclusion of predicting variables. The probability for removal was 0.10. Ocular ischaemia was used as the dependent variable: 0 = absent, 1 = present. Predicting variables were: age in years; sex: 0 = female, 1= male; two or more systemic symptoms, temporal artery palpable changes: 0 = absent, 1 = present; either halo counts or Halo Scores: 0 = below the cut-off value, 1 = equal to, or above the cut-off value. Nagelkerke R^2 and the model Chi Square (χ^2) were reported. Linearity was examined by the Box-Tidwell Transformation Test. Multicollinearity was examined as mentioned above.

P values <0.05 were considered statistically significant. Data were analysed with IBM SPSS Statistics 25, StatsDirect 3.1.22 and Graphpad Prism 5.

References

1. Hunder GG, Bloch DA, Michel BA, Stevens MB, Arend WP, Calabrese LH, et al. The American College of Rheumatology 1990 criteria for the classification of giant cell arteritis. Arthritis and Rheumatism 1990 Aug;33(8):1122-1128.

2. Luqmani R, Lee E, Singh S, Gillett M, Schmidt WA, Bradburn M, et al. The Role of Ultrasound Compared to Biopsy of Temporal Arteries in the Diagnosis and Treatment of Giant Cell Arteritis (TABUL): a diagnostic accuracy and cost-effectiveness study. Health technology assessment (Winchester, England) 2016 Nov;20(90):1-238.
