# Supplementary material for: Novel ultrasonographic Halo Score for giant cell arteritis: assessment of diagnostic accuracy and association with ocular ischaemia
Source: Ann Rheum Dis. 2020 Jan 3;79(3):393–9. doi: 10.1136/annrheumdis-2019-216343 (PMC7034352; doi:10.1136/annrheumdis-2019-216343)
Supplement: Supplementary data [file annrheumdis-2019-216343supp002.pdf]

Supplementary Tables

Supplementary Table S1. Alternative diagnoses in patients without GCA. The final diagnoses are shown for the 31 patients without a clinical diagnosis of GCA after 6 months follow-up. AION = anterior ischaemic optic neuropathy.

Final diagnosis	No. of patients (n=31)
Non-specific headache	10 (32%)
Sinusitis	3 (10%)
Migraine	2 (6%)
Temporomandibular disfunction	2 (6%)
Granulomatosis with polyangiitis	1 (3%)
Eosinophilic granulomatosis with polyangiitis	1 (3%)
Cerebral vasculitis	1 (3%)
Retinal vasculitis	1 (3%)
Thrombo-embolic event	1 (3%)
Optic neuritis	1 (3%)
Polymyalgia rheumatica	1 (3%)
Acoustic neuroma	1 (3%)
Cervical muscular spasm	1 (3%)
Non-arteritic AION	1 (3%)
Non-specific headache and sinusitis	1 (3%)
Non-specific headache and myofascial pain	1 (3%)
Myofascial pain, cervical spondylosis and fibromyalgia	1 (3%)
No clear alternative diagnosis	1 (3%)

van der Geest KSM, *et al. Ann Rheum Dis* 2020; 79:393–399. doi: 10.1136/annrheumdis-2019-216343

Supplementary Table S2. Halo thickness in the temporal artery segments and axillary arteries. Findings are based on baseline ultrasound examination of temporal and axillary arteries of 58 patients with an eventual clinical diagnosis of GCA after 6 months follow-up. Minimum/maximum values and percentiles of halo thickness are shown. A halo sign, i.e. a dark hypoechoic area around the vessel lumen, was present in 41 common superficial temporal artery (TA) segments, 29 parietal TA segments, 32 frontal TA segments and 14 axillary arteries.

	**Common superficial TA halo thickness (mm)**	**Parietal TA halo thickness (mm)**	**Frontal TA halo thickness (mm)**	**Axillary artery halo thickness (mm)**
Minimum	0.3	0.1	0.1	0.5
20% Percentile	0.4	0.3	0.2	0.6
40% Percentile	0.5	0.4	0.3	0.7
60% Percentile	0.6	0.4	0.4	0.9
80% Percentile	0.8	0.6	0.5	1.6
Maximum	1.4	1.0	0.9	6.7

van der Geest KSM, *et al. Ann Rheum Dis* 2020; 79:393–399. doi: 10.1136/annrheumdis-2019-216343

Supplementary Table S3. Halo grading in temporal arteries of GCA patients. Of the 58 patients with an eventual clinical diagnosis of GCA after 6 months follow-up, 45 patients showed at least one temporal artery halo at baseline. A halo sign was morphologically defined as an ultrasound finding of a dark hypoechoic area around the vessel lumen. The number of patients with at least one grade 1, 2, 3 or 4 temporal artery halo is shown.

Temporal artery halo grade	No. of patients with temporal artery halo fulfilling morphological criterium	
	**Halo absent (n=13)**	**Halo present (n=45)**
≥ 1 halo, grade 1 or more	0 (0%)	45 (100%)
≥ 1 halo, grade 2 or more	0 (0%)	39 (87%)
≥ 1 halo, grade 3 or more	0 (0%)	27 (60%)
≥ 1 halo, grade 4 or more	0 (0%)	14 (31%)

van der Geest KSM, *et al. Ann Rheum Dis* 2020; 79:393–399. doi: 10.1136/annrheumdis-2019-216343

Supplementary Table S4. Halo grading in axillary arteries of GCA patients. Of the 58 patients with an eventual clinical diagnosis of GCA after 6 months follow-up, 11 patients showed at least one halo on axillary artery ultrasound at baseline. A halo sign was morphologically defined as an ultrasound finding of a dark hypoechoic area around the vessel lumen. The number of patients with at least one grade 1, 2, 3 or 4 halo is shown. In addition, the number of patients with a halo thickness of 1.0 mm or more is shown [1].

**Axillary artery halo grade**	**No. of patients with axillary artery halo fulfilling morphological criterium**	
	Halo absent (n=47)	*≥ 1 halo (n=11)*
≥ 1 halo, grade 1 or more	0 (0%)	11 (100%)
≥ 1 halo, grade 2 or more	0 (0%)	8 (73%)
≥ 1 halo, grade 3 or more	0 (0%)	5 (46%)
≥ 1 halo, grade 4 or more	0 (0%)	3 (27%)
≥ 1 halo, 1.0 mm or more	0 (0%)	4 (36%)

Supplementary Table S5. Characteristics of non-GCA patients with a high halo count. Additional information regarding the two non-GCA patients with a high halo count is shown.

**Characteristics**	**Patient EP-12-033**	**Patient EP-12-037**
Clinical diagnosis	No clear diagnosis	Sinusitis
Sex	Female	Male
Halo count	6	5
Halo Score	11	6
≥ 1 halo cranial/axillary, grade 2 or more	Yes	Yes
≥ 1 halo cranial/axillary, grade 3 or more	No	No
Temporal artery biopsy finding	Intimal atherosclerosis	Normal

van der Geest KSM, *et al. Ann Rheum Dis* 2020; 79:393–399. doi: 10.1136/annrheumdis-2019-216343

Supplemental Table S6. Diagnostic accuracy of temporal artery ultrasound parameters for clinical diagnosis of GCA and temporal artery biopsy result. For the temporal artery halo count and temporal artery Halo Score, only measurements in the temporal artery segments were taken into account. Halo grading was similar to that described in Figure 1C. Data were obtained from the same subjects as shown in Figure 2. Area under the curve (AUC) obtained by ROC analysis is shown. TA = temporal artery. 95% CI = 95% confidence interval.

Reference standard	TA ultrasound parameter	AUC in ROC analysis (95% CI)
Clinical diagnosis after 6 months	TA halo count	0.702 (0.587-0.817)
	TA Halo Score	0.723 (0.611-0.834)
Temporal artery biopsy	TA halo count	0.821 (0.729-0.912)
	TA Halo Score	0.837 (0.747-0.927)

van der Geest KSM, *et al. Ann Rheum Dis* 2020; 79:393–399. doi: 10.1136/annrheumdis-2019-216343

Supplementary Table S7. Diagnostic accuracy of baseline halo count and Halo Score at the 95% specificity cut-off point. Subjects included all 58 patients with an eventual clinical diagnosis of GCA and 31 patients without a clinical diagnosis of GCA. The 95% specificity cut-off points for the halo count and Halo Score were a count of ≥6 and score of ≥10, respectively. LR+ = positive likelihood ratio. LR- = negative likelihood ratio.

Ultrasound finding	No. patients with GCA (% of total)	No. patients without GCA (% of total)	Sensitivity	Specificity	LR+	LR-
Halo count ≥ 6	2 (3%)	1 (3%)	3%	97%	1.07	1.00
Halo Score ≥ 10	12 (21%)	1 (3%)	21%	97%	6.41	0.82

van der Geest KSM, *et al. Ann Rheum Dis* 2020; 79:393–399. doi: 10.1136/annrheumdis-2019-216343

Supplementary Table S8. Temporal artery biopsy result and halo grade. Data are shown for 58 patients with an eventual clinical diagnosis of GCA after 6 months follow-up. A halo sign was morphologically defined as an ultrasound finding of a dark hypoechoic area around the vessel lumen. The number of patients with at least one grade 1, 2, 3 or 4 halo is shown for patients with a positive (n=26) and negative (n=32) temporal artery biopsy.

**Arterial ultrasound**	**Halo**	**Temporal artery biopsy**	
		**Positive (n=26)**	**Negative (n=32)**
Temporal artery	≥ 1 halo, morphological	25 (96%)	20 (63%)
	≥ 1 halo, grade 1 or more	25 (96%)	20 (63%)
	≥ 1 halo, grade 2 or more	22 (85%)	17 (53%)
	≥ 1 halo, grade 3 or more	18 (69%)	9 (28%)
	≥ 1 halo, grade 4 or more	12 (46%)	2 (6%)
Axillary artery	≥ 1 halo, morphological	7 (27%)	4 (13%)
	≥ 1 halo, grade 1 or more	7 (27%)	4 (13%)
	≥ 1 halo, grade 2 or more	5 (19%)	3 (9%)
	≥ 1 halo, grade 3 or more	4 (15%)	1 (3%)
	≥ 1 halo, grade 4 or more	3 (12%)	0 (0%)

van der Geest KSM, *et al. Ann Rheum Dis* 2020; 79:393–399. doi: 10.1136/annrheumdis-2019-216343

Supplementary Table S9. Characteristics of GCA patients divided by glucocorticoid use prior to ultrasound. Details of the 58 patients with an eventual clinical diagnosis of GCA after 6 months follow-up. CRP was measured before initiation of high dose glucocorticoid treatment in 41 patients. Haemoglobin levels and platelet counts were determined prior to high dose glucocorticoid treatment or within 7 days after initiation of this treatment in 58 patients. AION = anterior ischaemic optic neuropathy. PION = posterior ischaemic optic neuropathy. RAPD = relative afferent pupillary defect. Statistical significance was tested by the Chi-squared test or Kruskal-Wallis test. No statistically significant differences were found.

**Patient characteristics**	**High dose GC use before US**		
	**0-1 days (n=26)**	**2-3 days (n=17)**	**4-7 days (n=15)**
Sex, no. of males	8 (31%)	4 (24%)	3 (20%)
Polymyalgia	6 (23%)	2 (12%)	6 (40%)
Ocular ischaemia (AION/PION/RAPD)	3 (12%)	4 (24%)	5 (33%)
CRP, mg/L, median (range)	58 (3-234)	50 (13-206)	43 (27-329)
Haemoglobin, (g/dL), median (range)	11.9 (9.7-15.0)	12.1 (10.3-14.4)	12.2 (8.9-15.5)
Platelets, 10^9/L, median (range)	392 (167-627)	343 (184-661)	366 (175-636)

van der Geest KSM, *et al. Ann Rheum Dis* 2020; 79:393–399. doi: 10.1136/annrheumdis-2019-216343

Supplemental Table S10. Correlations between temporal artery ultrasound parameters and systemic inflammation. For the temporal artery halo count and temporal artery Halo Score, only measurements in the temporal artery segments were taken into account. Halo grading was similar to that described in Figure 1C. Data were obtained from the same subjects as shown in Figure 3. CRP levels were determined prior to initiation of treatment in 41 GCA patients. Haemoglobin levels and platelet counts were measured prior to treatment or within 7 days after initiation of high dose glucocorticoids in 58 GCA patients. Correlations were determined by Spearman's rank correlation coefficient.

**Laboratory test**		**TA halo count**	**TA Halo Score**
CRP	Spearman's rho	0.273	0.373
	p value	0.084	0.016
Haemoglobin	Spearman's rho	-0.167	-0.225
	p value	0.211	0.089
Platelets	Spearman's rho	0.240	0.216
	p value	0.069	0.103

van der Geest KSM, *et al. Ann Rheum Dis* 2020; 79:393–399. doi: 10.1136/annrheumdis-2019-216343

Supplementary Table S11. Axillary artery involvement and systemic inflammation. Data are based on 58 patients with an eventual clinical diagnosis of GCA after 6 months follow-up. CRP levels, haemoglobin levels and platelet counts are shown for patients with and without an axillary artery halo sign. CRP levels were determined prior to initiation of treatment in 41 GCA patients. Haemoglobin levels and platelet counts were measured prior to treatment or within 7 days after initiation of high dose glucocorticoids in 58 GCA patients. A halo was morphologically defined as an ultrasound finding of a dark hypoechoic area around the vessel lumen. In addition, the number of patients with a halo thickness of 1.0 mm or more is shown [1]. Statistical significance was tested by Mann-Whitney U test: * $p < 0.05$.

**Laboratory test**	**Axillary artery halo fulfilling morphological criterium**		**Axillary artery halo with thickness of 1.0 mm or more**	
	**No halo (n=47)**	**≥ 1 halo (n=11)**	**No halo (n=54)**	**≥ 1 halo (n=4)**
CRP, mg/L, median (range)	52 (3-329)	91 (12-234)	52 (3-329)	91 (60-206)
Haemoglobin, g/dL, median (range)	12.1 (8.9-15.5)	11.7 (9.7-12.8)	12.1 (8.9-15.5)*	11.0 (9.7-11.7)*
Platelets, 10^9/L, median (range)	361 (167-661)	366 (224-636)	363 (167-661)	453 (224-627)

van der Geest KSM, *et al. Ann Rheum Dis* 2020; 79:393–399. doi: 10.1136/annrheumdis-2019-216343

Supplementary Table S12. ESR and CRP levels in patients with GCA. Pre-treatment ESR was measured in 39 patients with an eventual clinical diagnosis of GCA after 6 months follow-up, and pre-treatment CRP levels in 41 GCA patients. The ESR was measured by a capillary photometric-kinetic technique (Alifax) [2].

Laboratory test	Value	No. of patients (%)
ESR	> 50 mm/hr	18 (46%)
	< 30 mm/hr	12 (31%)
CRP	> 40 mg/L	25 (61%)
	< 30 mg/L	9 (22%)
	< 20 mg/L	4 (10%)
	< 10 mg/L	1 (2%)

van der Geest KSM, *et al. Ann Rheum Dis* 2020; 79:393–399. doi: 10.1136/annrheumdis-2019-216343

Supplementary Table S13. Axillary artery involvement and ocular ischaemia. Data are based on 58 patients with an eventual clinical diagnosis of GCA after 6 months follow-up. Presence of ocular ischaemia is shown for patients with and without an axillary artery halo sign. Ocular ischaemia was defined as presence of anterior ischaemic optic neuropathy (AION), posterior ischaemic optic neuropathy (PION) and/or a relative afferent pupillary defect (RAPD). A halo was morphologically defined as an ultrasound finding of a dark hypoechoic area around the vessel lumen. In addition, the number of patients with a halo thickness of 1.0 mm or more is shown [1]. Statistical significance was tested by Fisher's exact test. No statistically significant differences were found.

**Characteristic**	**Axillary artery halo fulfilling morphological criterium**		**Axillary artery halo with thickness of 1.0 mm or more**	
	**No halo (n=47)**	**≥ 1 halo (n=11)**	**No halo (n=54)**	**≥ 1 halo (n=4)**
Ocular ischaemia, no. of patients (%)	9 (19%)	3 (27%)	11 (20%)	1 (25%)

van der Geest KSM, *et al. Ann Rheum Dis* 2020; 79:393–399. doi: 10.1136/annrheumdis-2019-216343

Supplementary Table S14. Variables predicting ocular ischaemia. Logistic regression analysis assessing either halo counts or Halo Scores, and other patient characteristics, as predictors for ocular ischaemia. Ocular ischaemia was defined as presence of anterior ischaemic optic neuropathy (AION), posterior ischaemic optic neuropathy (PION) and/or a relative afferent pupillary defect (RAPD). Halo count and Halo Score cut-off values were obtained by the ROC analysis and Youden Index, as shown in Figure 4. Binary logistic regression analysis was performed with backward exclusion of predicting variables. The probability of removal was 0.10. Results of the final model are shown. Age in years. Sex: 0 = female, 1= male. Ocular ischaemia, two or more systemic symptoms (i.e. anorexia, fever/night sweats, fatigue), temporal artery palpable changes (i.e. thickening and/or loss of pulse): 0 = absent, 1 = present. (-) Variable removed due to backward exclusion. 95% CI = 95% confidence interval.[a] R^2 (Nagelkerke) = 0.219. $\chi^{2(1)} = 8.731$, $p = 0.003$. [b] R^2 (Nagelkerke) = 0.238. $\chi^{2(2)} = 9.564$, $p = 0.008$.

**Dependent variable**	**Predicting variable**	**Final model, Odds ratio (95% CI)**	***p* value**
Ocular ischaemia	Age	-	
	Sex	-	
	Polymyalgia	-	
	Two or more systemic symptoms	-	
	Temporal artery palpable changes	-	
	Baseline halo count ≥ 2	12.000 (1.430-100.705)[a]	0.022
Ocular ischaemia	Age	1.071 (0.984-1.167)[b]	0.112
	Sex	-	
	Polymyalgia	-	
	Two or more systemic symptoms	-	
	Temporal artery palpable changes	-	
	Baseline Halo Score ≥ 3	9.880 (1.137-85.887)[b]	0.038

van der Geest KSM, *et al. Ann Rheum Dis* 2020; 79:393–399. doi: 10.1136/annrheumdis-2019-216343

Supplementary Figures

Supplementary Figure S1. Effect of short-term high dose glucocorticoids on halo count and Halo Score. (A) Baseline halo count and (B) Halo Score in patients with an eventual clinical diagnosis of GCA, who were on high glucocorticoid (GC) dose for 0-1 days (n=26; including patients that were not yet taking glucocorticoids), 2-3 days (n=17) or 4-7 days (n=15) prior to the baseline ultrasound examination. GC = glucocorticoid. US = ultrasound. Statistical significance was tested by the Kruskal-Wallis test. No statistically significant differences were found.

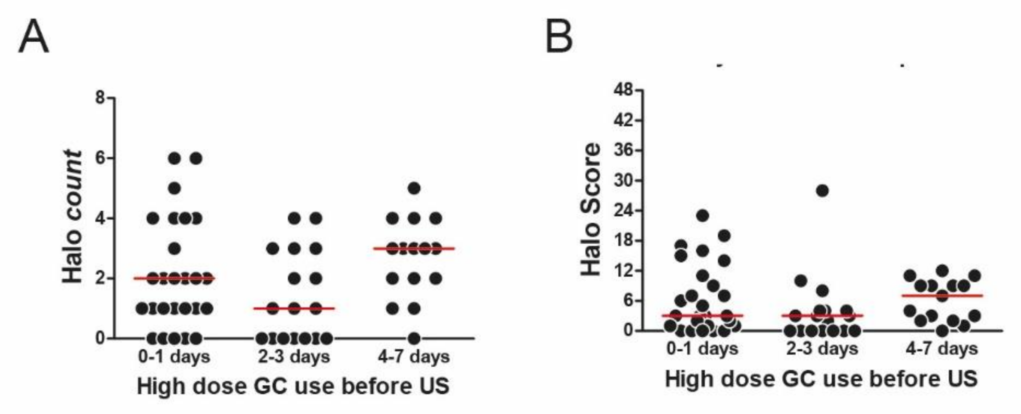

Supplementary Figure S2. No correlation between ESR and ultrasound parameters or CRP levels. The ESR and CRP levels were measured prior to initiation of high dose glucocorticoid treatment. (A) No correlation between the ESR and halo counts in patients with an eventual clinical diagnosis of GCA (n=39). (B) No correlation between the ESR and Halo Scores in patients with GCA (n=39). (C) No correlation between the ESR and CRP levels in patients with GCA (n=33). Correlations were determined by Spearman's rank correlation coefficient.

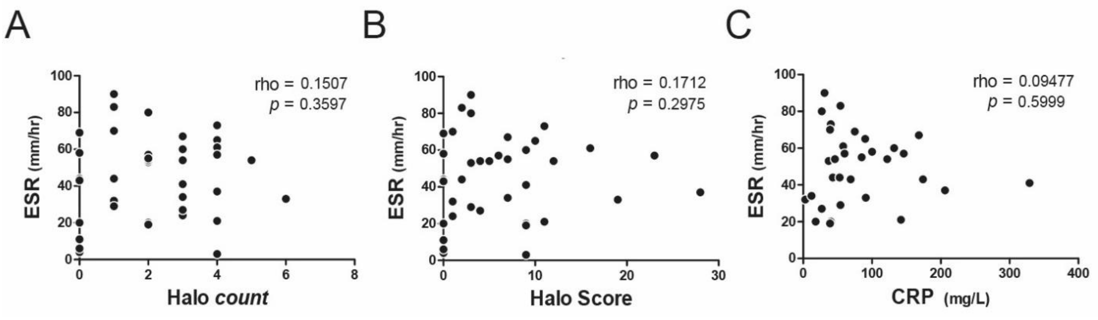

van der Geest KSM, *et al. Ann Rheum Dis* 2020; 79:393–399. doi: 10.1136/annrheumdis-2019-216343

Supplemental Figure S3. Temporal artery halo count and temporal artery Halo Score associated with risk of ocular ischaemia. For the temporal artery halo count and temporal artery Halo Score, only measurements in the temporal artery segments were taken into account. Halo grading was similar to that described in Figure 1C. (A) ROC curve showing diagnostic accuracy of baseline temporal artery halo count (left panel) and temporal artery Halo Score (right panel) for concomitant presence of ocular ischaemic symptoms. Ocular ischaemia was defined as presence of anterior ischaemic optic neuropathy (AION), posterior ischaemic optic neuropathy (PION) and/or a relative afferent pupillary defect (RAPD). The optimal cut-off points were determined by Youden Index. (B) Presence of ocular ischaemia (percentages are shown) among patients with low versus high temporal artery halo count (left panel), or low versus high temporal artery Halo Score (right panel) as determined by the optimal cut-off points mentioned at (A). AUC = area under the curve. Sens = sensitivity. Spec = specificity. LR+ = positive likelihood ratio. LR- = negative likelihood ratio.

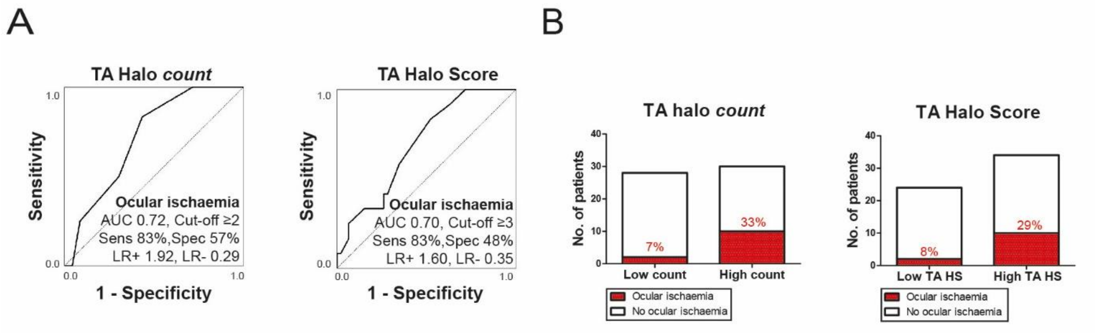

van der Geest KSM, *et al. Ann Rheum Dis* 2020; 79:393–399. doi: 10.1136/annrheumdis-2019-216343

References

1. Schafer VS, Juche A, Ramiro S, Krause A, Schmidt WA. Ultrasound cut-off values for intima-media thickness of temporal, facial and axillary arteries in giant cell arteritis. Rheumatology (Oxford, England) 2017 Sep 1;56(9):1632.

2. Kratz A, Plebani M, Peng M, Lee YK, McCafferty R, Machin SJ, et al. ICSH recommendations for modified and alternate methods measuring the erythrocyte sedimentation rate. International journal of laboratory hematology 2017 Oct;39(5):448-457.

van der Geest KSM, *et al. Ann Rheum Dis* 2020; 79:393–399. doi: 10.1136/annrheumdis-2019-216343
